# Development of a Duplex dPCR Assay for Detecting Palm Lethal Yellowing Phytoplasmas in Africa and Madagascar and Separation of Regional Species by High-Resolution Melt Curve Analysis (HRMA) Based on the *secA* Gene

**DOI:** 10.3390/biology14091175

**Published:** 2025-09-02

**Authors:** Melody Bloch, Fabian Pilet, Ericka E. Helmick, Mbolarinosy R. Rakotomalala, Brian W. Bahder

**Affiliations:** 1 Department of Entomology and Nematology & Department of Plant Pathology, Fort Lauderdale Research and Education Center, University of Florida, Gainesville, FL 33314, USA; mbloch@ufl.edu (M.B.); ehelmick@ufl.edu (E.E.H.); 2 CIRAD, UMR PVBMT, F-97410 Saint-Pierre, La Réunion, France; fabian.pilet@cirad.fr; 3 CIRAD, UMR PHIM, F-97170 Petit-Bourg, Guadeloupe, France; 4 Direction Générale du FOFIFA, Antananarivo 101, Madagascar; mbolarinosy@yahoo.fr

**Keywords:** diagnostic, quantitative PCR, digital PCR, survey, pathogen, plants

## Abstract

Palm lethal yellowing phytoplasmas (PLYPs) are a group of phytoplasmas that cause death in infected hosts across the tropics. Historically, detection and identification has relied on standard PCR, nested PCR, and restriction fragment length polymorphism. While these approaches are generally good, they are prone to error and contamination that is significantly lower or absent in modern approaches using quantitative PCR (qPCR) and digital PCR (dPCR). Additionally, these modern approaches are more time-efficient and consume fewer resources, making them more cost-effective in the long term. Recent studies have adapted dPCR and qPCR coupled with high-resolution melt curve analysis (HRMA) for PLYPs in the Caribbean/New World; however, these tools have not been developed for phytoplasmas in Africa and Madagascar. In this study, a duplex dPCR assay was developed with two specific assays, one for ‘*Candidatus* Phytoplasma palmicola’ and one for ‘*Ca.* P. cocostanzaniae’ and isolates from Madagascar. Additionally, primers targeting the *secA* gene were optimized and allowed for the separation of ‘*Ca.* P. cocostanzaniae’ and Malagasy isolates by approximately one degree Celsius. New primers were developed based on *secA* for ‘*Ca.* P. palmicola’ that allowed for the separation of the two subgroups (A and B) by HRMA by a difference of approximately one degree Celsius. These assays provide a valuable resource to explore aspects such as vector discovery and host range.

## Simple Summary

Palm lethal phytoplasmas cause severe losses to a variety of palm species in the tropics. In Africa and Madagascar, various strains cause decline in coconut palms. Currently, there are no specific, advanced molecular diagnostic tools that would allow for rapid and accurate means to confirm infection and study ecological aspects of the disease necessary for management. In this study, various tests were developed and optimized that allow for the rapid detection and identification of these phytoplasmas without sequencing or restriction profiles.

## 1. Introduction

Palm lethal yellowing phytoplasmas (PLYPs) are a unique group of pathogens that cause death in infected hosts (*Arecaceae* spp.). Symptoms associated with these pathogens are commonly referred to as lethal yellowing type syndromes (LYTSs) [1]. While PLYP-associated LYTSs are pantropical, there are three primary geographical groups of PLYPs, with the first occurring in the Neotropics, ranging from as far north as Georgia, the U.S.A. [2], west to Texas, the U.S.A. [3], and south to Guadeloupe in the Caribbean [4]. Within this region, there are three documented species: ‘*Candidatus* Phytoplasma palmae’, which causes the disease known as lethal yellowing (LY) and is the most widespread and abundant species in the region [5]; ‘*Ca.* P. aculeata’, which causes the disease known as lethal bronzing (LB) and is currently the predominant species associated with palm decline in the U.S.A. [6] and Mexico [7]; and finally, ‘*Ca.* P. hispanola’, a species that is closely related to ‘*Ca.* P. palmae’ and was originally discovered in the Dominican Republic [8] with one report from Mexico [9]. The second geographical group is in Africa (including Madagascar); there are at least two different phytoplasmas affecting coconut: ‘*Ca.* P. palmicola’ [10], with two distinct subgroups 16SrXXII-A and –B, with 16SrXXII-A being the subgroup present in East Africa [11]; ‘*Ca.* P. cocostanzaniae’, which is distributed in Tanzania and Mozambique [12,13]; and a recently discovered phytoplasma related to ‘*Ca.* P. cocostanzaniae, from western Madagascar [14]. The third geographical group comprises two species in the Australasian region: ‘*Ca.* P. noviguineense’ in Papua New Guinea [15] and ‘*Ca.* P. dypsidis’ in Australia [16].

In the respective ranges, the primary host of these phytoplasmas is the coconut palm (*Cocos nucifera* L.). In the New World, ‘*Ca.* P. hispanola’ appears to exclusively infect coconut. While ‘*Ca.* P. palmae’ is more virulent in coconut (apparently the most susceptible palm species), it has been documented from a variety of ornamental palms that are not native to the region [17]. Conversely, LB rarely infects coconut and predominantly infects the cabbage palm (*Sabal palmetto*) and date palms (*Phoenix* spp.) [6]. Generally, the symptoms of PLYPs in palms across hosts, phytoplasma species, and regions are similar, with an initial latency period where the phytoplasma is present in the palms without expressing symptoms. The latency period can vary considerably, from being entirely absent to up to four months [6]. Following the latent period, primary symptoms are premature fruit drop and/or the necrosis of the inflorescences, followed by the premature senescence of the most mature fronds that progresses to younger fronds, death of the apical meristem causing the collapse of the spear leaf, and finally, death of the palm. Despite this commonality in symptoms, there is both significant variation in the color of symptomatic leaves across host and phytoplasma species as well as geographical variation. Furthermore, the rate of decline from symptom onset also varies significantly, and even the same palm species infected with the same phytoplasma infecting both individuals adjacent to each other can vary significantly, from dying as quickly as three to four weeks after symptom onset to persisting up to eight months [18]. There are undoubtedly many variables that influence symptom variability across each host and region as it pertains to phytoplasma species interaction that likely include, but are not limited to, the general health of the palm, age, soil conditions, the plant microbiome, the number of infective insects inoculating the palm, the location in the canopy where infection occurs, and exposure to direct sun or wind.

The rapid detection and identification of corresponding phytoplasma species responsible for LYTS is critical not only for monitoring and management efforts but also for basic research as it pertains to phytoplasma biology [19,20] and vector discovery [21]. The increased sensitivity, accuracy, and time efficiency of quantitative PCR (qPCR) makes it far more practical than nested PCR techniques, especially when qPCR assays can be adapted to digital PCR (dPCR) systems. Moreover, the differentiation of the phytoplasmas affecting coconut palms in East Africa currently requires an additional step of restricting PCR products with several enzymes, further driving up the costs and potential source of error while not being as reliable as qPCR or dPCR assays. Recently, a specific TaqMan assay based on the 16S rRNA gene has been developed for the New World PLYPs that amplify all three species [9] and, in addition, high-resolution melt curve analysis (HRMA) has been utilized to rapidly differentiate the New World PLYPs using the *secA* gene [22]. These assays are routinely used in diagnostic services that provide stakeholders in Florida, U.S.A., where both ‘*Ca.* P. palmae’ and ‘*Ca.* P. acuelata’ are present, with practical data for management programs and have also been adapted to dPCR systems that aided in the discovery of *Haplaxius crudus* Van Duzee as the vector of LB in Florida [21]. To date, no specific qPCR, dPCR, or HRMA assay has been developed for African PLYPs.

The primary objective of this study was to develop TaqMan assays specific to the PLYPs present in Africa and Madagascar and to separate these phytoplasmas based on HRMA. With the recent discovery of PLYPs in Madagascar [14] and the initiation of vector surveys there, there is a need for a cost- and time-effective assay to evaluate plant and insect samples for the presence of phytoplasma. By developing assays for all known PLYPs in the East African region, standardized protocols can be available for research across the region to both clearly address research needs as well as supplement management efforts. Furthermore, it will allow for a more efficient means to evaluate changes in the distribution of each respective phytoplasma species and aid in determining if there are invasion events in mainland Africa and Madagascar or vice versa that result because of the commercial exchange between the two landmasses.

## 2. Materials and Methods

### 2.1. Phytoplasma Isolates, DNA Extraction, and Verification of Identity

**Figure 1:**
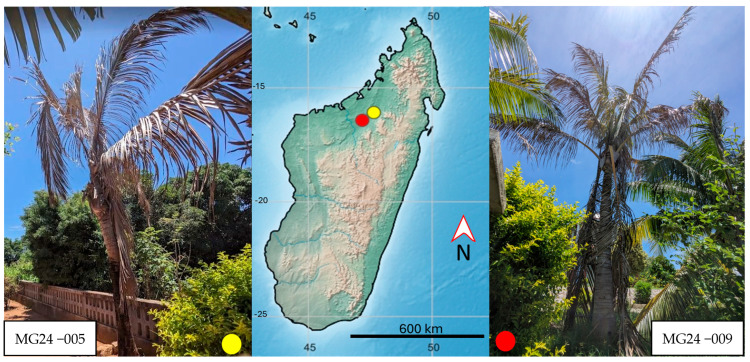
Declining coconut palms infected with phytoplasma in northwestern Madagascar that served as source material for molecular assay development and optimization.

Isolates of ‘*Ca.* P. palmicola’ (16SrXXII-A and 16SrXXII-B) and ‘*Ca.* P. cocostanzaniae’ maintained at the University of Florida’s Fort Lauderdale Research and Education Center (FLREC) were used as templates for generation of the corresponding sequence data and assay design (Table 1). The phytoplasma isolates from Madagascar were collected from late January to early February 2024 from symptomatic coconut palms (Figure 1) in the towns of Ambondromamy (isolate MG24−009) and Mampikony (isolate MG24−005) (northwestern Madagascar) (Table 1) (Figure 1) with permission of the owners. Each palm trunk was surface sterilized with bleach, pseudobark was removed, and an electric drill was used to obtain approximately 5 g of internal trunk tissue collected in 50 mL falcon tubes with paper towels and silica gel packets at the bottom to prevent mold growth. Total DNA was extracted from trunk tissue using a modified protocol of the DNeasy Plant Mini Kit (Qiagen, Germantown, MD, USA). Modification involved maceration of 1 g of trunk tissue in a BioReba extraction bag with approximately 5 mL of GTC buffer (guanidine thiocyanate, 4 M; sodium acetate, 3 M; EDTA, 0.5 M; PVP-40) using a HOMEX6 tissue homogenizer. Homogenate (400 µL) was transferred to a 2.0 mL microcentrifuge tube and processed according to the manufacturer’s instructions. All isolates were screened using the *secA* primers and protocol outline in [23]. Amplicons of the expected size were purified using the Exo-SAP-IT^TM^ PCR Product Cleanup Reagent (ThermoFisher Scientific, Waltham, MA, USA). The purified PCR product was quantified using a NanoDrop Lite spectrophotometer (ThermoFisher Scientific, Waltham, MA, USA) and Sanger sequenced on a SeqStudio Genetic Analyzer (Applied Biosystems, ThermoFisher Scientific). The resulting forward and reverse sequences were assembled, trimmed, and cleaned using DNA Baser (Version 4.36) (Heracle BioSoft SRL, Pitesti, Romania) and aligned using Clustal*W* as part of the package MEGA12 [24]. Sequences were submitted to a BLASTn search to confirm identity.

### 2.2. Assay Design and Optimization

#### 2.2.1. Oligonucleotides

*secA* sequence files for the above-mentioned isolates were analyzed using OligoArchitect (Sigma-Aldrich, St. Louis, MO, USA) for designing TaqMan assays. Assays with an overall quality rating of 80 or above, with no secondary structures or primer-dimers, were selected (Table 2). Primers for *secA*-based HRMA from [22] (Table 2) were used for ‘*Ca.* P. cocostanzaniae’ and the Malagasy strain but did not function for ‘*Ca.* P. palmicola’. Primers for utilization in HRMA were designed visually from aligned sequences to ensure regions with significant variability among isolates were incorporated into the target region for analysis for ‘*Ca.* P. palmicola’ isolates (Table 2).

#### 2.2.2. Gradient PCR for Optimal Annealing Temperature

Resulting HRMA and TaqMan assay primers were evaluated by standard gradient PCR to determine optimal annealing temperatures. Reactions comprised 5 µL 5× GoTaq Flexi Buffer, 2.5 µL 25 mM MgCl2, 0.5 µL 10 mM dNTPs, 0.5 µL of each 10 μM primer, 5 µL 10% PVP-40, 0.2 µL 2.5 U GoTaq Flexi DNA Polymerase, 2 μL DNA template, and sterile dH_2_O to a final volume of 25 μL. Thermal cycling conditions were as follows: initial denaturation at 95 °C for 2 min followed by 34 cycles of denaturation at 95 °C for 30 s, gradient annealing (48 to 58 °C) for 30 s, and extension at 72 °C for 10 s. Products were visualized using a 1.5% agarose gel stained with GelRed (Biotium, Hayward, CA, USA).

#### 2.2.3. Cloning for Standard Generation

Amplicons from the gradient PCR for each phytoplasma strain and corresponding primer sets were cloned using the pGEM-T Easy Vector kit (Promega) following the manufacturer’s instructions. Cloned vectors were transformed into NEB Turbo Competent *E. coli* (New England BioLabs) and plated on Lysogeny broth (LB) plates containing 100 µg/mL of ampicillin (Alkali Scientific, Ft. Lauderdale, FL, USA), 20 µg/mL X-Gal, and 16 µg/mL IPTG in solution (AG Scientific, San Diego, CA, USA). Plates were incubated at 37 °C overnight and transformed colonies were screened for clones with correct inserts using M13F/M13R primers in a standard PCR reaction: 2 min at 94 °C for initial denaturation followed by 34 cycles of denaturation at 94 °C for 30 s, annealing at 55 °C for 30 s, and extension at 72 °C for 1 min 30 s, followed by final extension at 72 °C for 10 min. PCR products were run on a 1.5% agarose gel stained with GelRed (Biotium, Hayward, CA, USA) and visualized under ultraviolet light. Clones with an insert of the correct size were incubated at 37 °C overnight on a shaker at 250 rpm in 20 mL of LB broth containing 100 mg/mL of ampicillin (Alkali Scientific, Ft. Lauderdale, FL, USA). Plasmids were extracted using a QIA-prep Spin Miniprep Kit (Qiagen) per the manufacturer’s instructions and sent for Sanger sequencing (Eurofins Genomics) to confirm identity of the inserts. Plasmid eluate was subsequently diluted to 10^10^ copies/μL followed by a serial dilution to 10^1^ copies/μL. For ‘*Ca.* P. palmicola’ (subgroup A), a synthetic control was generated for the corresponding region to generate the standard curve (Twist Biosciences, San Francisco, CA, USA).

#### 2.2.4. TaqMan Assay Optimization

For TaqMan optimization by qPCR, assays were performed on a QuantStudio 6 Flex thermal cycler (Life Technologies, Carlsbad, CA, USA). Plasmid dilutions and synthetic controls for ‘*Ca.* P. cocostanzaniae’, ‘*Ca.* P. palmicola’, and the unknown Madagascar strain were run in triplicate by qPCR to generate standard curves. Additionally, total DNA samples (∼25 ng/μL) of all African PLYPs were tested in triplicate. Negative controls including DNA extracted from healthy palm tissue and water control were also run in triplicate. Reactions comprised 10.3 μL of TaqMan Universal Master Mix II, with UNG (ThermoFisher Scientific, Waltham, MA, USA), 0.3 μL of 10 μM forward primer, 0.3 μL of 10 μM reverse primer, 0.6 μL of the 10 μM probe, 4 μL 10% PVP-40 (Polyvinylpyrrolidone), 1 μL DNA template, and sterile dH_2_O to a final volume of 20 μL. Thermal cycling conditions were as follows: hold stage 50 °C for 2 min, then ramp up at 1.6 °C/s to 95 °C for 10 min, followed by 35 cycles of denaturation at 95 °C for 15 s ramp down at 1.6 °C/s to annealing/extension at 54 °C for 1 min.

For duplex optimization by digital PCR (dPCR), assays were performed on a QuantStudio™ Absolute Q™ Digital PCR System (ThermoFisher Scientific). Total DNA samples (∼25 ng/μL) of all African PLYPs were tested. Additionally, the following mixed samples were generated by combining DNA extracts of each African PLYP: ‘*Ca.* P. cocostanzaniae’ + ‘*Ca.* P. palmicola’-A (C+A), ‘*Ca.* P. cocostanzaniae’ + ‘*Ca.* P. palmicola’-B (C+B), Malagasy + ‘*Ca.* P. palmicola’-A (M+A) and Malagasy + ‘*Ca.* P. palmicola’-B (M+B). These were subsequently tested using dPCR. Reactions comprised 1.8 μL Absolute Q Master Mix (5x), 0.3 μL of each 10 μM forward primer, 0.3 μL of each 10 μM reverse primer, 0.6 μL of each 10 μM probe, 1 μL DNA template (0.5 μL of each isolate that was diluted to 0.25 ng/µL), and sterile dH_2_O to a final volume of 9 μL. Thermal cycling conditions were as follows: preheat 95 °C for 10 min followed by 35 cycles of denaturation at 95 °C for 15 s and annealing/extension at 53 °C for 1 min.

#### 2.2.5. HRMA Assay Optimization

For HRMA assay optimization by qPCR, plasmid dilutions for ‘*Ca.* P. cocostanzaniae’, ‘*Ca.* P. palmicola’, and the Malagasy strain were run in triplicate by qPCR to assess amplification efficiency and HRMA. Total DNA samples (∼25 ng/μL) of all African PLYPs and negative controls were tested in triplicate. Reactions comprised 10 μL MeltDoctor HRM Master Mix, 3 μL of 10% PVP-40 (Polyvinylpyrrolidone), 1.2 μL of 5 μM forward pimer, 1.2 μL of 5 μM reverse primer, 1 μL of DNA template, and sterile dH_2_O to a final volume of 20 μL. Thermal cycling conditions were as follows: initial denaturation 95 °C for 10 min followed by 35 cycles of denaturation 95 °C for 15 s and annealing/extension at 55 °C for 30 s. The ramp rate for all steps was 1.6 °C/s. The conditions for HRMA were as follows: 95 °C for 10 s, 60 °C for 1 min, ramp phase at 0.1 °C/s using step-and-hold function at 5 s, and dissociation at 95 °C for 15 s. Aligned melt curves and difference plots were generated using the HRMA application (ThermoFisher).

## 3. Results

### 3.1. TaqMan Assays

Two separate TaqMan assays were developed. One assay meeting quality standards was designed for ‘*Ca.* P. cocostanzaniae’ and the Malagasy strain (FAM labeled) and one assay meeting quality standards was designed for ‘*Ca.* P. palmicola’ (VIC labeled) (Table 2), both with a 3’ NFQ-MGB quencher. For the *secA* gene, there was no sufficient variation between ‘*Ca.* P. cocostanzaniae’ and the Malagasy strain at sites acceptable for assay designed that would have allow for strain-specific assays.

**Figure 2:**
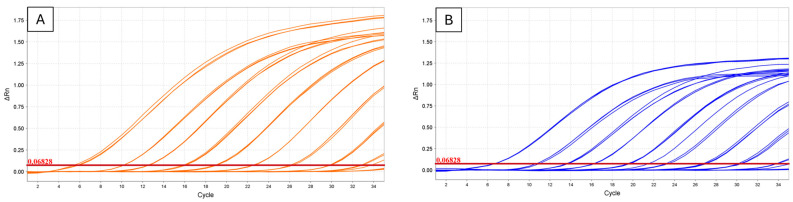
Plasmid standards with the *secA* insert for the Malagasy species (**A**) and ‘*Ca.* P. cocostanzaniae’ (**B**) screened with the corresponding TaqMan assay.

**Figure 3:**
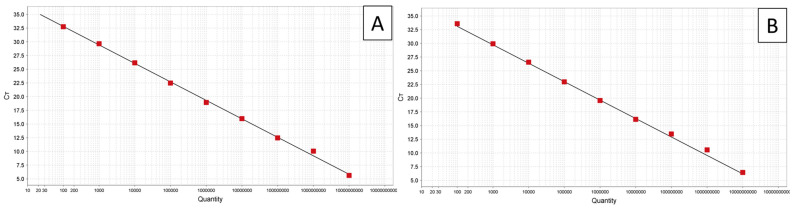
Standard curve generated from plasmids with *secA* inserts for ‘*Ca.* P. cocostanzaniae’ (**A**) and the Malagasy species (**B**) serially diluted from 10^9^ copies/µL to 10^1^ copies/µL.

Plasmid standards with the *secA* insert for either ‘*Ca.* P. cocostanzaniae’ or the Malagasy strain were successfully amplified, except for those with 10^1^ copies/μL (Table 3, Figure 2). The optimal annealing was determined to be 54 °C for both assays (single, clear band in gradient PCR). The resulting standard curve for ‘*Ca.* P. cocostanzaniae’ had a slope of −3.371, *Y*-intercept of 39.543, *R*^2^ = 0.998, and efficiency of 98% with an error of 0.031 (Figure 3). The resulting standard curve for the unknown Madagascar strain had a slope of −3.361, *Y*-intercept of 40.136, *R*^2^ = 0.995, and efficiency of 98.407% with an error of 0.048 (Figure 3). When screened against the total DNA samples, ‘*Ca.* P. cocostanzaniae’ had an average Ct of 26.7 (SE ± 0.5) while the Malagasy strain had an average Ct of 30.4 (SE ± 0.0). The estimated titers for ‘*Ca.* P. cocostanzaniae’ and the Malagasy strain were 9002 (SE ± 3760) and 597 (SE ± 51), respectively. The other African PLYPs (‘*Ca.* P. palmicola) and the negative controls failed to be amplified in all reactions (Table 4).

**Figure 4:**
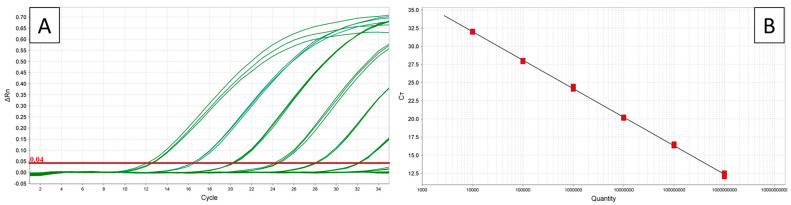
Amplification plot of dilution series of synthetic control with *secA* insert for ‘*Ca.* P. palmicola’-A (**A**) and corresponding standard curve (**B**).

Plasmid standards with the *secA* insert for ‘*Ca.* P. palmicola’ were successfully amplified, except for those with 10^3^ copies/μL or fewer (Table 3, Figure 4). The optimal annealing was determined to be 54 °C for both assays (single, clear band in gradient PCR). The resulting standard curve for ‘*Ca.* P. palmicola’ had a slope of −3.924, *Y*-intercept of 47.695, *R*^2^ = 0.999, and efficiency of 80% with an error of 0.023 (Figure 4). When screened against total DNA samples, ‘*Ca.* P. palmicola’-A had an average Ct of 31.5 (SE ± 0.0) while ‘*Ca.* P. palmicola’-B had an average Ct of 33.1 (SE ± 0.1). The estimated titers for ‘*Ca.* P. palmicola’-A and ‘*Ca.* P. palmicola’-B were 13,080 (SE ± 294) and 5189 (SE ± 319), respectively. The other African PLYPs (‘*Ca.* P. cocostanzaniae’ and Malagasy strain) and the negative controls failed to be amplified in all reactions (Table 4).

**Figure 5:**
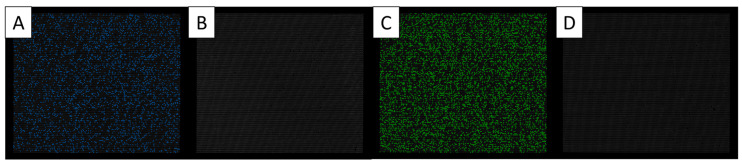
Duplex dPCR results; (**A**) ‘*Ca.* P. cocostanzaniae’ isolate with FAM labeled assay displaying positive FAM signal (blue), (**B**) ‘*Ca.* P. palmicola’ isolate with FAM labeled assay displaying no FAM signal, (**C**) ‘*Ca.* P. palmicola’ isolate with VIC labeled assay displaying VIC signal (green), and (**D**) ‘*Ca.* P. cocostanzaniae’ isolate with VIC labeled assay displaying no VIC signal.

**Figure 6:**
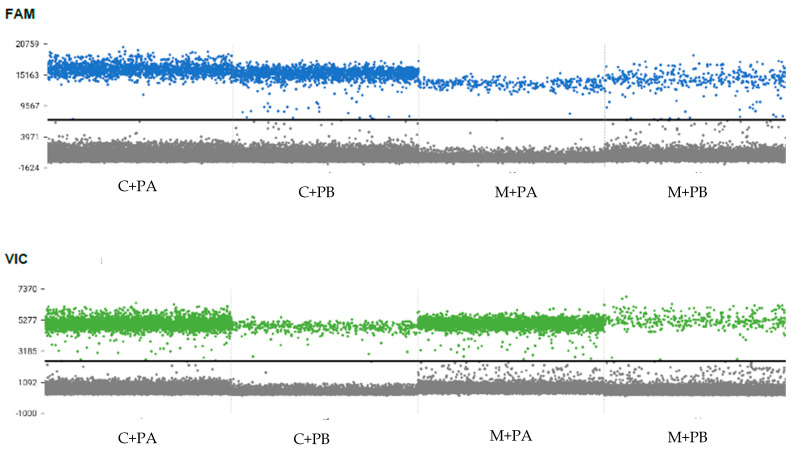
Amplification plots for the FAM and VIC channels for experimental mixtures of ‘*Ca.* P. cocostanzaniae’ (C), ‘*Ca.* P. palmicola’ subgroups A (PA) and B (PB), and the Malagasy isolates (M); black line = florescence threshold for scoring positive reactions.

The duplex dPCR assay successfully amplified all four phytoplasmas targeted in this study and no cross-amplification among the ‘*Ca.* P. palmicola’ assay and ‘*Ca.* P. cocostanzaniae’/Malagasy assay was detected (Figure 5). In experimental mixed infections of ‘*Ca.* P. cocostanzaniae’ + ‘*Ca.* P. palmicola’-A (C+A), ‘*Ca.* P. cocostanzaniae’ + ‘*Ca.* P. palmicola’-B (C+B), Malagasy + ‘*Ca.* P. palmicola’-A (M+A), and Malagasy + ‘*Ca.* P. palmicola’-B (M+B), both phytoplasmas were successfully detected each time (Figure 6, Table 5).

### 3.2. HRMA Assays

**Figure 7:**
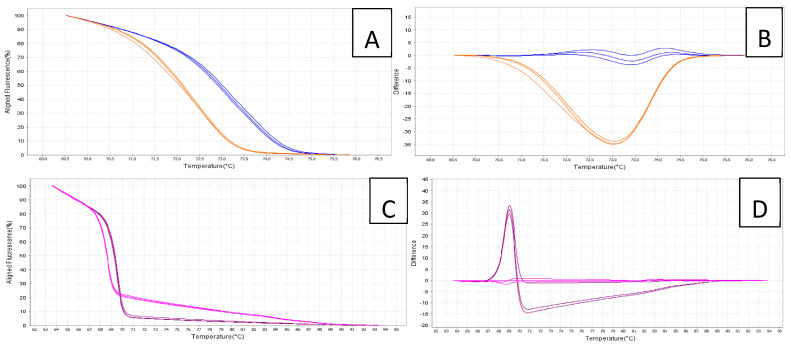
High-resolution melt curve analysis for ‘*Ca.* P. cocostanzaniae’ (orange) and Malagasy isolates (blue) displaying aligned curve (**A**) and difference plot (**B**) and for ‘*Ca.* P. palmicola’ strains A (purple) and B (pink) displaying aligned curves (**C**) and difference plot (**D**).

Plasmid dilutions with the *secA* insert ‘*Ca.* P. cocostanzaniae’ and the Malagasy strain were successfully amplified, except for those with 10^1^ copies/μL (Table 6, Figure 7) with the primers from Bloch et al. [22]. Changes in Ct due to dilution factors were consistent from 10^10^ to 10^2^ copies/µL. The Tm for ‘*Ca.* P. cocostanzaniae’ generally ranged from 72.3 to 72.4 °C at higher concentrations and increased to 72.8 to 73.0 °C at lower concentrations. For the Malagasy strain, the Tm ranged from 73.1 to 73.3 °C at higher concentrations, with higher variability at the lower concentrations.

When screened against the total DNA samples, ‘*Ca.* P. cocostanzaniae’ had an average Ct of 26.8 (SE ± 0.2) while the Malagasy strain had an average Ct of 30.3 (SE ± 0.2). The estimated Tm values for ‘*Ca.* P. cocostanzaniae’ and the Malagasy strain were 72.6 °C (SE ± 0.05) and 73.7 °C (SE ± 0.05), respectively (Table 7). The difference of approximately 1.1 °C in Tm for the amplicons of ‘*Ca.* P. cocostanzaniae’ and the unknown Madagascar strain produced significantly different melt curves, distinguishable on both the aligned melt curve and difference plot (Figure 7). The isolates of ‘*Ca.* P. palmicola’ and the negative controls failed to amplify in all reactions and produced Tm products not matching the positive controls.

The new primers for ‘*Ca.* P. palmicola’ (Table 2) successfully amplified plasmid dilutions with the *secA* insert for ‘*Ca.* P. palmicola’ subgroups 16SrXXII-A and 16SrXXII-B except for those with 10^1^ copies/μL (Table 6). Changes in Ct due to dilution factors were consistent from 10^10^ to 10^2^ copies/µL. The Tm for 16SrXXII-A generally ranged from 69.4 to 69.7 °C for all dilutions. For 16SrXXII-B, the Tm ranged from 68.5 to 68.7 °C for all dilutions. When screened against the total DNA samples, 16SrXXII-A had an average Ct of 27.5 (SE ± 0.1) while 16SrXXII-B had an average Ct of 27.7 (SE ± 0.6). The estimated Tm values for 16SrXXII-A and 16SrXXII-B were 69.6 °C (SE ± 0.02) and 68.7 °C (SE ± 0.02), respectively (Table 7). The difference of approximately 0.9 °C in Tm for the amplicons of 16SrXXII-A and 16SrXXII-B produced significantly different melt curves, distinguishable on both the aligned melt curve and derivative melt curves (Figure 7). The other isolates for ‘*Ca.* P. cocostanzaniae’, the Malagasy strain, and the negative controls failed to be amplify in all reactions for this primer set and produced Tm products not matching the positive controls.

**Table 5:** dPCR data for duplex assay on laboratory generated mixed infections of different combinations of the three phytoplasmas analyzed in this study.

	Species Mixture			
Target	C+PA ^1^	C+PB ^2^	M+PA ^3^	M+PB ^4^
Total Wells	20,473	20,465	20,465	20,457
FAM (C/M)				
Positive Wells	1750	1754	323	396
% Positive	8.6%	8.6%	1.6%	1.9%
Conc. (cp/µL)	206.84	207.42	36.83	45.25
95% C.I.	9.471, 9.925	9.487, 9.941	3.805, 4.243	4.244, 4.684
Precision %	4.798	4.793	11.523	10.351
VIC (A/B)				
Positive Wells	3063	430	2962	381
% Positive	15.0%	2.1%	14.5%	1.9%
Conc. (copies/µL)	375.14	49.16	361.91	43.52
95% C.I.	13.067, 13.539	4.433, 4.873	12.814, 13.285	4.158, 4, 597
Precision %	3.609	9.913	3.671	10.563

**Table 6:** HRMA data for plasmid dilutions of all phytoplasmas analyzed in this study.

	HRMA Assay							
	‘*Ca.* P. Palmicola’				‘*Ca.* P. Cocostanzaniae’/Malagasy Isolate			
Conc. (copies/µL)	Subgroup A		Subgroup B		‘*Ca.* P. Cocostanzaniae’		Malagasy Isolate	
	Avg. Ct (±SE)	Avg. Tm (±SE)	Avg. Ct (±SE)	Avg. Tm (±SE)	Avg. Ct (±SE)	Avg. Tm (±SE)	Avg. Ct (±SE)	Avg. Tm (±SE)
10^10^	6 ± 0.1	69.6 ± 0.01	4.9 ± 0.1	68.6 ± 0.00	5.8 ± 0.3	72.4 ± 0.05	5.9 ± 0.2	73.2 ± 0.05
10^9^	9.3 ± 0.0	69.5 ± 0.02	7.5 ± 0.1	68.6 ± 0.02	8.5 ± 0.2	72.4 ± 0.02	9.1 ± 0.4	73.3 ± 0.02
10^8^	12.3 ± 0.1	69.5 ± 0.03	9.8 ± 0.0	68.6 ± 0.02	11.1 ± 0.2	72.4 ± 0.04	11.6 ± 0.3	73.2 ± 0.04
10^7^	15.5 ± 0.0	69.7 ± 0.01	13.6 ± 0.1	68.7 ± 0.01	14.6 ± 0.1	72.4 ± 0.04	15.0 ± 0.2	73.2 ± 0.04
10^6^	19.1 ± 0.0	69.6 ± 0.02	17.2 ± 0.1	68.6 ± 0.02	18.4 ± 0.2	72.3 ± 0.03	18.9 ± 0.2	73.2 ± 0.03
10^5^	22.7 ± 0.0	69.4 ± 0.06	20.5 ± 0.1	68.6 ± 0.01	21.8 ± 0.1	72.3 ± 0.02	22.1 ± 0.2	73.1 ± 0.02
10^4^	26.1 ± 0.1	69.5 ± 0.01	23.9 ± 0.1	68.6 ± 0.02	25.7 ± 0.1	72.4 ± 0.04	25.8 ± 0.3	73.1 ± 0.04
10^3^	29.3 ± 0.3	69.5 ± 0.02	27.2 ± 0.0	68.6 ± 0.00	29.7 ± 0.9	72.8 ± 0.09	31.7 ± 1.2	73.7 ± 0.09
10^2^	33.1 ± 0.0	69.5 ± 0.03	30.9 ± 0.1	68.5 ± 0.00	32 ± 0.1	72.9 ± 0.05	32.2 ± 0.4	73.8 ± 0.05
10^1^	-	69.4 ± 0.08	34.1 ± 0.2	68.6 ± 0.00	-	73 ± 0.22	31.5 ± 2.1	73.2 ± 0.22

**Table 7:** HRMA data for DNA samples/isolates of all phytoplasmas analyzed in this study.

	HRMA Assay			
	‘*Ca.* P. Palmicola’		secA614F/secA759R	
	Avg. Ct (±SE)	Avg. Tm (±SE)	Avg. Ct (±SE)	Avg. Tm (±SE)
‘*Ca.* P. palmicola’ A				
Awka	27.5 ± 0.1	69.6 ± 0.02	No Ct	87.7 ± 6.82
Nig 1	26.7	69.6	No Ct	87.2
Nig 2	24.6	69.6	No Ct	73.7
185	21.4	69.6	No Ct	73.1
‘*Ca.* P. palmicola’ B				
ADN 22	27.7 ± 0.6	68.7 ± 0.02	No Ct	80.8 ± 7.92
ADN 19	28.5	68.7	No Ct	65.3
ADN 36	34.9	68.6	No Ct	63.7
‘*Ca.* P. cocostanzaniae’				
TT tall	No Ct	74.4 ± 4.73	26.8 ± 0.2	72.6 ± 0.05
EAT LY PS	No Ct	64.1	28.3	72.7
PB 121A	No Ct	65.3	34.2	73.0
Malagasy Isolates				
MG24−009	No Ct	78.2 ± 8.81	30.3 ± 0.2	73.7 ± 0.05
MG24−002	No Ct	68.7	28.4	73.2
MG24−005	No Ct	87.9	31.3	74.2
MG24−006	No Ct	63.2	28.8	73.4
MG24−007	No Ct	69.6	33.7	74.2

## 4. Discussion

The novel assays developed and optimized in this study represent the first utility of dPCR technology and HRMA on PLYPs in Africa and Madagascar. Because of the significantly higher level of sensitivity, accuracy and precision of these technologies over traditional, standard PCR and restriction fragment analysis combined with nested PCR, molecular diagnostics and research efforts on this group of pathogens in the region will greatly benefit. The development of a multiplex dPCR assay is a critical need for vector studies. Furthermore, with significantly higher level of accuracy of dPCR over qPCR due to the absolute quantification of single targets over estimating against a standard curve (as in qPCR), a better understanding of the titers and biology of these pathogens can be obtained. The vector of the PLYPs in Africa and Madagascar remains unknown.

Multiple studies have evaluated vectors of these phytoplasmas with no confirmed transmission to date. One study isolated phytoplasma from the derbid planthopper *Diostrombus mkurangai* and an unidentified species in the family Meenoplidae [25]. Additionally, a single specimen of *Diostrombus mayumbensis* was found positive by using nested PCR for phytoplasma in western Africa [26]. All studies, while confirming that the planthoppers are feeding on infected plants, have provided no evidence that they are competent vectors. Recently, the vector of LB was confirmed to be *Haplaxius crudus* [21] by the detection of the phytoplasma in the salivary glands and artificial feeding media, utilizing dPCR. The low titer of phytoplasma, both in the salivary glands and in the trace amounts transmitted to the feeding media, indicates that dPCR is a critical tool for rapid vector discovery (in both palm systems and likely beyond). The duplex assay in this study will be particularly useful for vector studies in east Africa where both ‘*Ca.* P. cocostanzaniae’ and ‘*Ca.* P. palmicola’ exist and overlap in range [27]. Any future vector studies will allow for the simultaneous detection and identification of the phytoplasma in target insect species collected from infected host plants. While this duplex assay can be used for palm diagnostic purposes, the level of sensitivity that dPCR generally is not necessary for detecting phytoplasma in palm tissue provided that symptoms are present; however, the low amounts observed in salivary glands and what is transmitted to artificial feeding media make it necessary for vector research as these levels are not detectable by standard PCR or even qPCR. Phytoplasmas infecting palms in Florida, the U.S.A. were found at high levels and fully systemic once symptoms appeared in *Sabal palmetto* and *Phoenix sylvestris* [20] and were also easily detectable three to four months prior to symptom onset [18] using qPCR. While the titer of phytoplasmas in infected palms in Africa and Madagascar has not been assessed, it seems unlikely that it would vary significantly to the point where qPCR would not be suitable. While quantity estimates were generated in this study using the duplex assay and dPCR, these were single data points generated from taking a sample from one location on the palm trunk. In *S. palmetto* and *P. sylvestris* infected with ‘*Ca.* P. aculeata’, it was demonstrated that the phytoplasma titer varied significantly both along the length of the trunk and even around the circumference at the same level and that this pattern shifted over time/with symptom progression [20]. With these novel assays, this study can be replicated on coconut in Africa and Madagascar to determine if phytoplasma titers and their distribution are similar to the trend observed in the New World. Because of this, the utility of HRMA coupled with qPCR is ideal for assessing palm infection status in the region because it is a time-efficient way to amplify and differentiate more closely related, yet distinct, species or strains that do not have sufficient variation for the development of specific TaqMan assays and yet may possess subtle ecological differences (i.e., host range, vector range).

The HRMA assay used in this study to differentiate ‘*Ca.* P. cocostanzaniae’ and isolates from Madagascar was the same developed by Bloch et al. [22], secA614F/secA759R. These primers failed to amplify ‘*Ca.* P. palmicola’ isolates so, unfortunately, could not be run congruently with the former phytoplasmas. While it was previously shown that ‘*Ca.* P. cocostanzaniae’ amplified with these primers and had a melt curve distinct from the three species found in the Caribbean, these are the first data showing that it was distinct from the phytoplasma found in Madagascar. Initially, the phytoplasma discovered in Madagascar was considered related to ‘*Ca.* P. cocostanzaniae’ based on the 16S rRNA gene [14]; however, a multilocus analysis suggested that it was distinct [28]. The difference in both the Tm product and melt curve showed that the difference between ‘*Ca.* P. cocostanzaniae’ and the Malagasy isolates (almost one degree Celsius) was larger than the difference between LY-associated ‘*Ca.* P. palmae’ and LB-associated ‘*Ca.* P. acuelata’ in the New World (differing by approximately a half degree Celsius). The current data based on separating the Malagasy isolates from ‘*Ca.* P. cocostanzaniae’ by the amount recorded further supports that the phytoplasmas in Madagascar and mainland Africa are distinct species. While ‘*Ca.* P. cocostanzaniae’ has a lower Tm product and melt curve compared to New World PLYPs, the isolates from Madagascar have a similar Tm product as ‘*Ca.* P. palmae’ and, ultimately, a similar melt curve. While these two phytoplasmas are certainly distinct species [28], the similarity of the melt curve and Tm product is a result of convergence and does not create problems from a diagnostic perspective because LY and the phytoplasmas in Madagascar are geographically separated and the likelihood of either of these phytoplasmas being introduced into the other’s range is low enough to be considered impossible from a practical perspective. While these assays worked well on the isolates analyzed in this study, there is undoubtedly much more genetic variation of these pathogens in nature. The limited sample size was based on material that was available and what was collected in the field, so it is possible that the differences in efficiency, utility, and Tm products/the melt curve may vary should more samples be included. However, with the development of these assays, it will be more cost-effective to study larger sample sizes and analyze their effectiveness in future studies.

## 5. Conclusions

The assays developed in this study represent valuable new tools that will allow for future research to better explore fundamental and applied aspects of these phytoplasmas’ epidemiology in the region. By having faster, more sensitive, and more cost-effective approaches, aspects such as vector discovery, palm host range, and reservoir identification can be conducted more effectively.

## Figures and Tables

**Table 1:** Phytoplasma isolates used in this study for the development of TaqMan and HRMA assays based on the *secA* gene.

Species	Isolate	Locality	GenBank Accession No.
‘*Ca.* P. palmicola’ (16SrXXII-A)	Awka	Nigeria	PX136635
	Nig1	Nigeria	PX136636
	Nig2	Nigeria	PX136637
	185	Nigeria	PX136638
‘*Ca.* P. palmicola’ (16SrXXII-B)	ADN22	Côte d’Ivoire	PX136639
	ADN19	Côte d’Ivoire	PX136640
	ADN36	Côte d’Ivoire	PX136641
‘*Ca.* P. cocostanzaniae’	TanzTagTall	Tanzania	PX136643
	EAT LY PS	Tanzania	PX136644
	PB 121A	Tanzania	PX136642
Malagasy isolates	MG24-002	Madagascar	PX136646
	MG24-005	Madagascar	PX136645
	MG24-006	Madagascar	PX136647
	MG24-007	Madagascar	PX136648
	MG24-009	Madagascar	PX136649

**Table 2:** Molecular assays designed in this study for detecting and differentiating African and Malagasy palm lethal phytoplasmas.

Assay Type	Species	Orientation	Sequence (5′-3′)	Annealing Temp.
TaqMan	‘*Ca.* P. cocostanzaniae’/ Malagasy isolate	Sense	CAGGAAGAATTTTGCATG	
		Antisense	CATCCTTCTTTAGCTTCTAA	54 °C
		Probe -Sense	FAM-ATGTAAACCATCGCTAAATTGACG-NFQ-MGB	
	‘*Ca.* P. palmicola’	Sense	CTCCTGATTTGATATTTGTTAA	
		Antisense	GCTGTACCAATTAAAATAGG	54 °C
		Probe—Antisense	VIC-TTGATGTCGGTCTTCTTATCTTCTAA-NFQ-MGB	
HRMA	‘*Ca.* P. palmicola’	Sense	TAGCCCTCAAAATTGTAA	54 °C
		Antisense	ACCAGTAAATTGATCTACA	
	‘*Ca.* P. cocostanzaniae’/Malagasy	Sense	GGWCGTCAATTTAGTGAWGG [22]	55 °C
		Antisense	GCMGTTCCTGTCATTCCTGA [22]	

**Table 3:** qPCR data for plasmid standard/synthetic control dilution series for phytoplasmas analyzed in this study.

	‘*Ca.* P. Cocostanzaniae’	Malagasy Isolate MG24-009	‘*Ca.* P. Palmicola’
Conc. (copies/µL)	Avg. Ct (±SE)	Avg. Ct (±SE)	Avg. Ct (±SE)
10^10^	5.6 ± 0.1	6.5 ± 0.0	Not assessed
10^9^	10.1 ± 0.0	10.7 ± 0.1	12.3 ± 0.2
10^8^	12.4 ± 0.0	13.5 ± 0.0	16.4 ± 0.1
10^7^	15.9 ± 0.1	16.1 ± 0.0	20.2 ± 0.0
10^6^	18.8 ± 0.1	19.5 ± 0.1	24.3 ± 0.1
10^5^	22.4 ± 0.0	22.5 ± 0.6	28.0 ± 0.1
10^4^	26 ± 0.1	26.8 ± 0.1	32.0 ± 0.0
10^3^	29.7 ± 0.0	30.1 ± 0.1	-
10^2^	33 ± 0.3	34.1 ± 0.4	-
10^1^	-	-	-

**Table 4:** qPCR data for corresponding assays on all isolates analyzed in this study.

		FAM (C/M)		VIC (PA/PB)	
Species	Isolate	Avg. Ct (±SE)	Qty. (copies/µL)	Avg. Ct (±SE)	Qty. (copies/µL)
‘*Ca.* P. palmicola’ subgroup A	Awka	No Ct	0.0	31.5 ± 0.0	13,080 ± 294
	Nig 1	No Ct	0.0	29.7 ± 0.0	38,075 ± 1018
	Nig 2	No Ct	0.0	28.9 ± 0.7	74,829 ± 33,145
	185	No Ct	0.0	23.1 ± 0.1	1,848,754 ± 130,683
‘*Ca.* P. palmicola’ subgroup B	ADN 22	No Ct	0.0	34.1 ± 0.1	2881 ± 105
	ADN 19	No Ct	0.0	33.1 ± 0.1	5189 ± 319
	ADN 32	No Ct	0.0	32.4 ± 0.1	7745 ± 309
‘*Ca.* P. cocostanzaniae’	TT tall	26.7 ± 0.5	9002 ± 3760	No Ct	0.0
	EAT LY PS	26.7 ± 0.1	8069 ± 632	No Ct	0.0
	PB 121A	32.1 ± 0.3	325 ± 43	No Ct	0.0
Malagasy isolates	MG24-002	25.03± 0.2	26137 ± 2249	No Ct	0.0
	MG24-005	29.2 ± 0.0	1468 ± 22	No Ct	0.0
	MG24-006	26.4 ± 0.1	15710 ± 517	No Ct	0.0
	MG24-007	30.7 ± 0.3	650 ± 87	No Ct	0.0
	MG24-009	30.4 ± 0.0	597 ± 51	No Ct	0.0

## Data Availability

All the data generated in this study is presented in the manuscript under the Results section. Sequences used to generate assays are publicly available in GenBank.

## Abbreviations

The following abbreviations have been used in this manuscript:

PLYP	Palm lethal yellow phytoplasmas
LYTS	Lethal yellowing type syndrome
dPCR	Digital PCR
qPCR	Quantitative PCR
HRMA	High-resolution melt curve analysis
LY	Lethal yellowing
LB	Lethal bronzing
